# Sarcoidosis in Patients with Psoriasis: A Population-Based Cohort Study

**DOI:** 10.1371/journal.pone.0109632

**Published:** 2014-10-06

**Authors:** Usman Khalid, Gunnar Hilmar Gislason, Peter Riis Hansen

**Affiliations:** 1 Department of Cardiology, Gentofte Hospital University of Copenhagen, Hellerup, Denmark; 2 Faculty of Health Sciences, University of Copenhagen, Copenhagen, Denmark; 3 National Institute of Public Health, University of Southren Denmark, Copenhagen, Denmark; Oregon Health & Science University, United States of America

## Abstract

**Purpose:**

Psoriasis is a chronic inflammatory disease characterized by a systemic immunological response which is mainly driven by activated T helper (Th) 1 and Th17 lymphocytes. Like psoriasis, sarcoidosis is a chronic inflammatory disorder with Th1/Th17-driven inflammation. Therefore, we investigated the risk of sarcoidosis in patients with psoriasis compared to the background population in a nationwide cohort.

**Methods:**

The study included the entire Danish population aged ≥10 years followed from 1^st^ January 1997 until diagnosis of sarcoidosis, death or 31^st^ December 2011. Patients with a history of psoriasis and/or sarcoidosis at baseline were excluded. Information on comorbidity and concomitant medication was identified by individual-level linkage of administrative registers. Incidence rates of sarcoidosis were calculated and adjusted hazard ratios (HRs) were estimated by multivariable Cox regression models adjusted for age, gender, comorbidity, medications and socioeconomic status.

**Results:**

A total of 6,043,518 subjects were eligible for analysis. In the study period 70,125 patients with new-onset psoriasis, including 11,834 patients with severe psoriasis, were identified. The overall incidence rates of sarcoidosis were 1.18, 2.22, and 4.06 per 10,000 person-years for the reference population (9,717 cases), mild psoriasis (78 cases) and severe psoriasis (22 cases), respectively. Compared to the reference population, the age- and gender-adjusted HRs for sarcoidosis were increased in patients with psoriasis with HR 1.49 (95% confidence interval [CI] 1.18–1.87) and HR 2.51 (CI 1.64–3.85) for those with mild and severe disease, respectively.

**Conclusion:**

In this nationwide cohort, psoriasis was associated with a disease severity-dependent increased risk of sarcoidosis.

## Introduction

Psoriasis is a chronic inflammatory disease characterized by a systemic immunological response which is mainly driven by activated T helper (Th) 1 and Th17 lymphocytes [1]; [2]. Increasing evidence has suggested that psoriasis is not just an isolated skin disease and it is associated with increased comorbidity, e.g. autoimmune disorders and cardiovascular disease, that may, in part, be due to shared inflammatory mechanisms [3]–[7]. Sarcoidosis is a chronic granulomatous disorder, usually presenting with pulmonary infiltrates, hilar lymphadenopathy and skin lesions. Although the disease aetiology is poorly understood, the role of Th1- and Th17-mediated inflammation in formation of sarcoidal granulomas is well documented [8]–[10]. In view of this apparent pathogenic link between the two diseases it is interesting that limited case series have been presented with concurrent sarcoidosis and psoriasis [11]–[13]. However, no large scale epidemiological data exist on this topic and we therefore examined the association between sarcoidosis and psoriasis, including the impact of psoriasis severity, in a nationwide cohort.

## Methods

### Data sources

The study was conducted and reported in accordance with cohort study guidelines outlined in the Strengthening the Reporting of Observational Studies in Epidemiology (STROBE) recommendations [14]. The permanent personal registration number assigned to each individual at birth in Denmark provides an exclusive possibility to integrate data from nationwide registers on an individual level. We used four of these registers to acquire data for the present cohort study. The Central Population Register comprises information on all individuals living in Denmark, registered with date of birth and gender. The Danish National Patient Register holds information on dates and causes of hospitalisations in Denmark, registered according to the international Classification of Diseases (ICD) system and recorded since 1978. The Danish National Prescription Register includes data on all dispensed prescription in Denmark, including information on dispensing date, quantity dispensed and the strength of the drug since 1995. Dispensed drugs are registered by Anatomical Therapeutic Classification (ATC) codes, and because of partial reimbursement of drug expenses, the accuracy of the register is very good [15]. In the National Causes of Deaths Register, all deaths in Denmark are registered within 2 weeks after occurrence. An age-standardized index of socioeconomic status from 0 to 4 was defined on the basis of individual annual income during a 5-year period before study start.

### Study population

The study cohort comprised entire Danish population aged ≥10 years, starting from 1^st^ January 1997 and followed until 31^st^ December 2011, diagnosis of sarcoidosis, migration or death. Psoriasis patients were identified by prescriptions claimed for topical vitamin D derivatives (ATC code D05AX), which is first-line treatment used exclusively for psoriasis and is not accessible without prescription in Denmark. To ensure persistence in medical treatment for psoriasis, patients were first included in the study when claiming their second prescription for these agents. Patients were classified as having severe psoriasis at the time of their third hospitalisation or out-patient consultation for psoriasis (ICD-10 L40) or psoriatic arthritis (M070–M073). We have previously validated this method for identification and classification of psoriasis [16]; [17]. Patients with a history of psoriasis and/or sarcoidosis were excluded from the study at baseline.

### Comorbidity and pharmacotherapy

Charlson comorbidity index was used for adjustment in all analyses and included all diagnoses at study entry and up to 1 year previously [18]. Baseline treatment was defined by dispensed prescriptions up to 6 months prior to study entry for the following drugs (ATC codes): glucocorticoids (H02AB), methotrexate (L01BA01), and anti-diabetic drugs (A10).

### Outcome

The study endpoint was a diagnosis of sarcoidosis (ICD-10 code D86 and ICD-8 code 135) recorded in the National Patient Registry.

### Statistical analysis

Baseline characteristics are presented as means with standard deviations or frequencies and percentages. Comorbidity and concomitant medications were considered as fixed variables at baseline. Psoriasis was included as a time-dependent variable and thus subjects that developed psoriasis contributed with risk time in the reference group until time of diagnosis. Incidence rates were calculated as events per 10,000 person-years at risk. Cox proportional hazards models were used to estimate hazard ratios (HRs) for the study endpoint. Age, gender, comorbidity, socioeconomic status, and concomitant medications were included as covariates in the analyses.

A two-tailed p value <0.05 was considered statistically significant and 95% confidence intervals (CIs) presented for all analyses. All statistical analyses were performed with the SAS statistical software version 9.2 (SAS Institute Inc. Cary, NC, USA) and STATA software version 11.0 (StataCorp, College Station, TX, USA).

## Results

### Baseline characteristics

**Figure 1:**
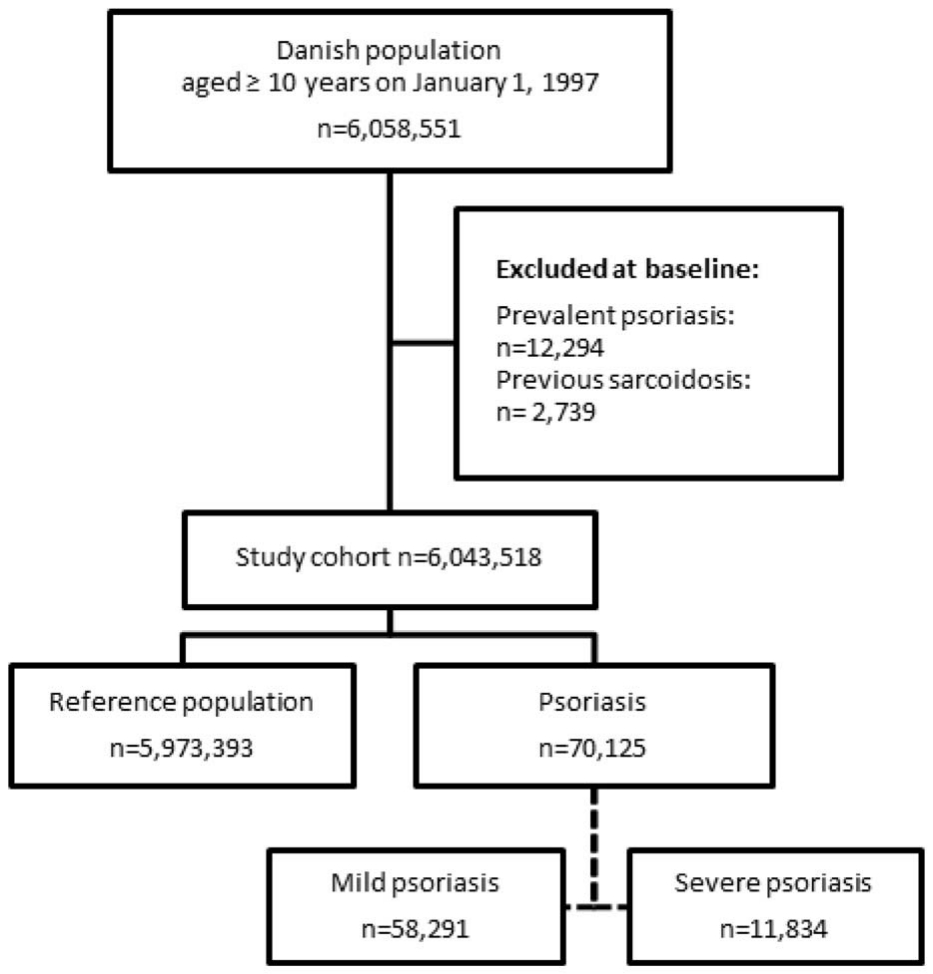
Flowchart of the selection of study population.

The study included a total of 6,058,551 subjects, aged ≥10 years. Subjects with a history of psoriasis (n = 12,294) and sarcoidosis (n = 2,739) were excluded from the analysis at baseline. A total of 58,291 subjects with mild psoriasis and 11,834 with severe psoriasis were identified during the study period. These patients were compared with the reference population of 5,973,393 individuals. A flowchart of the study population selection is shown in Figure 1. In comparison with the reference population, patients who developed severe psoriasis had a comparable comorbidity at baseline, but slightly increased use of methotrexate (Table 1).

**Table 1:** Baseline characteristics of the study population.

	Reference population (n = 5,973,393)	Mild psoriasis (n = 58,291)	Severe psoriasis (n = 11,834)
Age (mean [SD]) years	37.0 (21.8)	42.2 (18.3)	41.0 (16.6)
Male (%)	2,956,319 (49.5)	28,307 (48.6)	5,547 (47.0)
Follow-up time (mean [SD]) years	13.8 (3.2)	6.5 (4.3)	5.6 (3.7)
Socio-economic status (mean [SD])	2.0 (1.5)	2.5 (1.3)	2.5 (1.2)
Charlson’s index	0.03 (0.24)	0.02 (0.20)	0.03(0.27)
*Comorbidity (%)*			
Cerebrovascular disease	13,956 (0.2)	109 (0.2)	10 (0.1)
Cardiac dysrhythmia	15,251 (0.3)	125 (0.2)	34 (0.3)
Renal disease	2,447 (0.0)	12 (0.0)	6 (0.0)
Chronic obstructive pulmonary disease	12,284 (0.2)	68 (0.1)	21 (0.2)
Diabetes mellitus	14,579 (0.2)	147 (0.3)	44 (0.4)
Peripheral vascular disease	6,415 (0.1)	54 (0.1)	8 (0.1)
Previous myocardial infarction	7,757 (0.1)	80 (0.1)	13 (0.1)
Rheumatic disease	4,061 (0.1)	33 (0.1)	21 (0.2)
*Medications (%)*			
Systemic glucocorticoids	87,165 (1.5)	1,088 (1.9)	370 (3.2)
Methotrexate	3,874(0.1)	136 (0.2)	311 (2.6)
Anti-diabetic drugs	74,637 (1.3)	849 (1.5)	208 (1.8)

### Risk of sarcoidosis

The results showed a significant association between psoriasis and new-onset sarcoidosis (Table 2). The overall incidence rates per 10,000 person-years were 1.18 (CI 1.15–1.20), 2.22 (CI 1.78–2.77), and 4.06 (CI 2.67–6.17) for the reference population, patients with mild and severe psoriasis, respectively. The mean follow up time was 13.6, 6.53 and 5.61 years with 9717, 78, and 22 cases of sarcoidosis in the reference group, mild psoriasis and severe psoriasis, respectively.

**Table 2:** Incidence rates (IRs) with 95% confidence intervals (CIs) of sarcoidosis per 10,000 person-years and number of events.

	Reference population	Mild psoriasis	Severe psoriasis
Sarcoidosis (IR [CI])	1.18 (1.15–1.20)	2.22 (1.78–2.77)	4.06 (2.67–6.17)
Number of events (n)	9,717	78	22

The multivariable cox regression analyses adjusted for age and gender, confirmed increased HRs for sarcoidosis in patients with psoriasis compared to the reference population with HR 1.49 (CI 1.18–1.87) and HR 2.51 (CI 1.64–3.85) for mild and severe psoriasis, respectively. The HRs associated with psoriasis remained statistically significant in the fully adjusted models that controlled for age, gender, comorbidity, concomitant medications, and socioeconomic status (Table 3).

**Table 3:** Risk of sarcoidosis associated with psoriasis.

Sarcoidosis	Hazard ratio	95% confidence interval	p-value
*Adjusted for age and gender*			
Mild psoriasis	1.49	1.18–1.87	<0.001
Severe psoriasis	2.51	1.64–3.85	<0.001
*Adjusted for age, gender, comorbidity, medications and socioeconomic status*			
Mild psoriasis	1.40	1.11–1.76	<0.004
Severe psoriasis	2.33	1.52–3.57	<0.001

## Discussion

In this contemporary nationwide cohort study we examined the risk of sarcoidosis in patients with psoriasis compared to the general population. Analyses were adjusted for age, gender, concomitant medications, comorbidity and socioeconomic status, and the HRs for sarcoidosis were found to be significantly increased in patients with psoriasis compared to the general population. Importantly, this increased risk was disease severity-dependent.

Psoriasis is one of the most prevalent systemic inflammatory diseases, characterized by increased activity of Th1 and Th17 lymphocytes [1]; [2]. There is growing body of evidence that psoriasis along with other chronic inflammatory diseases has a higher prevalence of comorbid conditions which is may be due, in part, to shared immunoinflammatory pathways [3]–[7]. Sarcoidosis is a chronic disease of poorly understood etiology, mainly characterized by sarcoidal granulomas with of aggregates of Th1 and Th17 cells. Moreover, prior studies have demonstrated increased levels of interleukin (IL)-17 in bronchoalveolar lavage fluid and peripheral blood in these patients [9]. In view of the apparent overlap of immunoinflammatory mechanisms, interest in a possible correlation between psoriasis and sarcoidosis was recently rejuvenated after publication of the as yet largest case series consisting of 7 patients with concurrent psoriasis and sarcoidosis [11]–[13]. To the best of our knowledge, however, no large scale epidemiological study has previously been carried out to more definitively examine the association between these two diseases.

The present study demonstrated a strong association between psoriasis and sarcoidosis. Moreover, this association increased with increasing psoriasis severity and remained statistically significant after adjustments for potential confounding factors, which strongly supports the results and suggests existence of shared causal pathways. In this regard, coincident inflammatory mechanisms are likely to play a role and are probably dependent on shared susceptibility genes, e.g., polymorphisms in the IL-23 receptor gene albeit that at present, the weight of evidence for this particular genetic predisposition is considerably stronger for psoriasis than for sarcoidosis [19]–[21]. However, there may be other as yet undefined mechanisms, e.g. it is possible that in rare cases topical vitamin D analogues may induce hypercalcaemia which again can contribute to the sarcoidosis diagnosis [22]; [23]. Accordingly, additional studies are clearly warranted to delineate mechanisms underlying the association between psoriasis and sarcoidosis and potential clinical consequences of this phenomenon.

### Study strengths and limitations

The major strengths of the present study are the use of real-world nationwide data from a large unselected population, completeness of follow-up, adjustment for important confounders, and use of validated measures of exposure and diagnoses. Furthermore, use of nationwide registers of drug prescriptions and hospitalizations in Denmark where health care is essentially free of charge and equally accessible to all citizens, makes surveillance bias less likely. The possibility of selection bias related to, e.g., age, gender, health insurance and socioeconomic status is reduced by inclusion of the entire Danish population aged≥10 years. In addition, exclusion of subjects with prevalent psoriasis and/or sarcoidosis at study ensured precise allocation of time at risk for all subjects.

There are several important limitations to be acknowledged. The study was observational and therefore not capable of establishing causal mechanisms. The method used to identify patients with psoriasis based on dispensed prescriptions for vitamin D derivatives, i.e., first-line treatment for psoriasis in Denmark, does not account for patients receiving other topical psoriasis therapies, e.g., glucocorticoids. However, we have previously validated this approach and shown that vitamin D derivatives are used in approximately 3/4^th^ of patients that receive continuous topical treatment, and any bias related to potential misclassification is expected to favor the null hypothesis [16]; [17]. Along this line, we have also previously demonstrated that patients with psoriasis referred to hospitals generally present with a mean psoriasis area and severity index score of 10, i.e. compatible with severe psoriasis [16]; [17]. Furthermore, severe psoriasis was classified by number of hospitalizations or out-patient consultations, which may have decreased the threshold for detection of sarcoidosis and comorbidities in this patient group, albeit that multiple adjustments for cofounding variables including the Charlson comorbidity index were made to counter this limitation.

Sarcoidosis is likely to be underreported probably due to variations in diagnostic criteria, the wide spectrum of phenotypes, and frequent lack of definitive symptoms at disease onset [10]; [24]. Hence, sarcoidosis is often diagnosed secondarily to hospital visits for other and/or less defined reasons (including psoriasis) leading to detection bias. Furthermore, the registers used in the study do not hold information on important factors such as sarcoidosis phenotypes, e.g., pulmonary, cutaneous, cardiac and/or systemic disease, and clinical factors of potential relevance, e.g., smoking status and body weight. Rare cases have also been reported where treatment with tumor necrosis factor (TNF)-α inhibitors was associated with paradoxical development of sarcoidosis, usually in patients with rheumatologic diagnoses including psoriatic arthritis [25]; [26]. Currently, such treatment is not accurately captured in Danish registers and could therefore not be controlled for in our analyses, but not least in view of the paucity of reported cases in the literature where sarcoidosis was associated with treatment with TNF-α inhibitors, this phenomenon is unlikely to have considerably influenced our results. Finally, the population in Denmark is predominantly composed of Caucasians and there are geographical and ethnic variations in the incidences of sarcoidosis and psoriasis, which may limit the generalizability of the findings [27]; [28].

## Conclusion

This first nationwide study indicated a disease severity-dependent association between psoriasis and sarcoidosis. The results add to evidence that patients with psoriasis have increased risk of immunoinflammatory conditions. Additional studies are warranted to explore the mechanisms underlying the correlation between psoriasis and sarcoidosis and delineate the potential clinical consequences of this association.
